# Uterine Fibroid Patients Reveal Alterations in the Gut Microbiome

**DOI:** 10.3389/fcimb.2022.863594

**Published:** 2022-05-11

**Authors:** Xuetao Mao, Xuan Peng, Qiong Pan, Xingping Zhao, Zheng Yu, Dabao Xu

**Affiliations:** ^1^Department of Gynecology, The Third Xiangya Hospital, Central South University, Changsha, China; ^2^Department of Microbiology, School of Basic Medical Science, Central South University, Changsha, China

**Keywords:** gut microbiota, uterine fibroids, microbial interactions, 16S rRNA microarray, community diversity

## Abstract

The gut microbiota is associated with reproductive disorders in multiple ways. This research investigated possible differences in gut microbiome compositions between patients with uterine fibroids (UFs) and healthy control subjects in order to further provide new insight into its etiology. Stool samples were collected from 85 participants, including 42 UF patients (case group) and 43 control subjects (control group). The gut microbiota was examined with 16S rRNA quantitative arrays and bioinformatics analysis. The α-diversity in patients with UFs was significantly lower than that of healthy controls and negatively correlated with the number of tumorigeneses. The microbial composition of the UF patients deviated from the cluster of healthy controls. Stool samples from patients with UFs exhibited significant alterations in terms of multiple bacterial phyla, such as Firmicutes, Proteobacteria, Actinobacteria, and Verrucomicrobia. In differential abundance analysis, some bacteria species were shown to be downregulated (*e*.*g*., *Bifidobacteria scardovii*, *Ligilactobacillus saerimneri*, and *Lactococcus raffinolactis*) and upregulated (*e*.*g*., *Pseudomonas stutzeri* and *Prevotella amnii*). Furthermore, the microbial interactions and networks in UFs exhibited lower connectivity and complexity as well as higher clustering property compared to the controls. Taken together, it is possible that gut microbiota dysbiosis has the potential as a risk factor. This study found that UFs are associated with alterations of the gut microbiome diversity and community network connectivity. It provides a new direction to further explore the host–gut microbiota interplay and to develop management and prevention in UF pathogenesis.

## Introduction

Uterine fibroids (also known as leiomyomas or myomas) are the most common benign neoplasms of the uterus. It is estimated that women (in the USA) have an up-to-75% lifetime risk of developing uterine leiomyomas (Commandeur et al., 2015), a pathology characterized by substantial extracellular matrix (Stewart et al., 1994; Baird et al., 2003). Symptoms related to fibroids include abnormal uterine bleeding, pelvic pain, urinary frequency, and constipation, which vary with the size and location of the fibroids (Wallach et al., 1981). Chromosomal damage associated with parity was relatively overrepresented in uterine leiomyomas (Kuisma et al., 2021). In addition, fibroids have been associated with infertility and poor obstetrical outcomes due to the abnormal uterine cavity shape and the compression of the fallopian tube (Bajekal and Li, 2000; Coronado et al., 2000). Therefore, it imposes a considerable burden on women of reproductive age and on society as a whole (Marsh et al., 2018).

Imbalances in the gut microbiota have been widely reported to have complex associations with human health, specifically by immune responses and nutrient metabolism (Behary et al., 2021; Leyrolle et al., 2021). On one hand, the host local immune system as well as the gut barrier function is affected by altered microbial interactions. Alterations contribute to the disruption of the intestinal homeostasis and result in the development of several human diseases, including chronic infectious diseases, gastrointestinal diseases, metabolic diseases, and even malignant tumors (Zhu et al., 2018; Mouries et al., 2019; Sims et al., 2019; Oh et al., 2020). On the other hand, the human gut is colonized with a vast community of indigenous microorganisms that have co-evolved with the host in a symbiotic relationship and also can alter among populations depending on the host’s dietary habits, gender, ethnicity, geographical environment, health status, *etc*. (Ma et al., 2020; Wei et al., 2020; Dwiyanto et al., 2021). For these reasons, the gut microbiota is now considered a potential source of novel therapeutics and interventions to improve the health status. To date, our understanding of the composition and functions of the human gut microbiota and possible pathogenic mechanisms has increased exponentially. Gut dysbiosis plays an important role in multi-system diseases. A growing body of evidence points out a link between diet and common reproductive pathologies (*e*.*g*., polycystic ovary syndrome, infertility, endometriosis, and/or deregulated ovarian functions) (Skoracka et al., 2021). On one hand, we hypothesize that unhealthy diets lead to gut dysbiosis, which is related to the development of UFs. On the other hand, the uterine fibroid is a sex hormone-related disease. Moreover, the gut microbiota regulates the levels of sex hormones *via* interactions among its metabolites, the immune system, and chronic inflammation (He et al., 2021). However, there is still no study that clearly shows any abnormalities of the gut microbiota in UF patients as compared to the control subjects.

In this study, we applied 16S rRNA quantitative microarrays, a novel high-throughput microarray technology, rather than conventional culture-based techniques to compare the gut microbiology differences between healthy individuals and patients with UFs. Furthermore, we explored the potential correlation and deciphered the interplay between the gut microbiome and UFs.

## Materials and Methods

### Study Design and Sample Collection

The participants with UFs (*n* = 42) and the control participants (*n* = 43) were recruited at The Third Xiangya Hospital of Central South University from December 2020 to May 2021. The UF patients were diagnosed by the Gynecology Department of The Third Xiangya Hospital according to the clinical practices (Stewart, 2015). The exclusion criteria included severe chronic diseases (*e*.*g*., metabolic disorders, heart failure, cirrhosis, and gastrointestinal, neurological, and/or autoimmune diseases). Moreover, individuals with a history of probiotic interventions, diarrhea, and taking antibiotics or NSAIDs within 3 months before prior to collection were also excluded. None of the female subjects was premenarchal or postmenopausal.

This study was approved by the Ethics Committee of The Third Xiangya Hospital of Central South University and was conducted under the relevant guidelines and regulations (IRB number 22003). Written informed consent had been obtained from the participants before the research, and all samples and questionnaires were voluntary. All UF subjects retained stools at the time of the diagnosis of the disease without initiating any treatment. Moreover, all fecal samples were collected after menstruation. Fresh stool samples were collected in sampling tubes with the preservative solution and stored at -80°C until further processing.

### DNA Extraction and Labeling

Bacterial DNA was extracted from stool samples using the Stool DNA Extraction Kit (Halgen, Ltd.) according to the procedures described in the manufacturer’s instruction. Primers F44 (RGTTYGATYMTGGCTCAG) and R1543 (GGNTACCTTKTTACGACTT) were used to amplify the DNA of the V1–V9 regions of the 16S rRNA gene. Approximately 20–30 ng of the extracted DNA was used in a 50-µl PCR reaction under the following cycling conditions: 94°C for 3 min for an initial denaturing step; then, followed by 94°C for 30 s, 55°C for 30 s, 72°C for 60s, altogether for a total of 30 cycles; and followed by a final extension step of 72°C for 3 min. Agarose gel electrophoresis was then applied to check if the PCR amplification was successful. Finally, the PCR products were directly labeled using a DNA labeling kit (Halgen Ltd., Zhong Shan, China) and further processed for microarray hybridization.

### Microarray Hybridization

The human gut bacterial microarrays used were designed and manufactured by Halgen Ltd. The arrays use its proprietary oligo-array technology and cover more than 95% of the culturable gut microbial species found in different populations. Probes were selected from all the variable regions of bacterial 16S rRNA. The length of each probe was designed to be approximately 40 bp. The hybridization mixture consisted of 500 ng of Cy5-labeled test sample DNA and 50 ng of Cy3-labeled reference pool. Then, the hybridization buffer and the Cy3- and Cy5-labeled samples were added to a final volume of 150 µl, heated to 100°C for 5 min, and cooled on ice for 5 min. All hybridization mixtures were placed in a hybridization box and then hybridized at 37°C for 3.5 h in a hybridization oven. Finally, the slides were washed in 2× saline sodium citrate, 0.25% Triton X-100, 0.25% sodium dodecyl sulfate, and 1X Dye Protector for 15 min at 63°C. Then, the slides were rinsed in 1X Dye Protector until they were clear of water droplets after immediate withdrawal from the solution. The slides were immediately scanned using a dual-channel scanner.

### Data Analysis

The Cy5/Cy3 ratio measured by the respective channels was used to calculate the percentage of each microbial species, which is presented as the relative abundance value. R (v.4.1.2) was used in this study. Alpha diversity (α-diversity) and beta diversity (β-diversity) indices were analyzed to characterize species diversity within and among habitats respectively, to evaluate their overall diversity in an integrated manner. α-Diversity includes richness, Shannon–Wiener diversity, Gini–Simpson diversity (obtained by subtracting the value of the classical Simpson index from 1), and Pielou’s evenness. They were measured using the function diversity in the package “Vegan” based on unpumped flat OTU table; the beta diversity indices of the microeukaryotic communities were calculated using the function vegdist after data were pumped. ANOSIM was chosen to test for significance between groups (Wang et al., 2008). For non-metric multidimensional scaling (NMDS), stress less than 0.2 indicates that the results of the NMDS analysis were scientifically credible (Legendre and Legendre, 1998). The species composition at the phylum level between the two groups was visualized using the ggplot2 package. The DEseq2 package was used to analyze species difference and marker species (Topper et al., 2017). The differential expression matrix and the *P*-value matrix of species composition were obtained through the function DESeqDataSetFromMatrix. The significance level was *P <*0.05, and the absolute FoldChange value was greater than 2; the volcano map was drawn using the ggplot2 package. The coexistence network of the two groups was established based on Spearman correlation matrix and corrected by *P*-value matrix using the igraph package; the Benjamini and Hochberg false discovery rate was used to correct the *P*-value. Modules are divided according to the high intra-module connectivity and the low inter-module connectivity; the Spearman correlation coefficient and corrected *P*-values were 0.4 and 0.05, respectively (Yuan et al., 2021). Then, the bacteria coexistence network was constructed in Gephi software (https://gephi.org/). A random network with the same number of nodes and edges as the real network was constructed by using the erdos.renyi.game function (Erdos and Renyi, 1960). The classified information of species in network modules was presented using ggplot2. The redundancy analysis (RDA) of the effect of age, body mass index (BMI), and other body indices on the distribution of samples and the distribution of species was performed by ggplot2’s built-in vegan package.

SPSS (version 26.0) was also used in this study. Continuous data were reported by median with range (minimum–maximum) or mean ± standard deviation (SD) and were appropriately analyzed with Wilcoxon test or *t*-test. The categorical data were described with the number and percentages and were analyzed with *χ*^2^ test or Fisher’s exact test as appropriate. *P* < 0.05 was considered to be statistically significant.

## Results

### Clinical Characteristics of the Study Subjects

The demographic characteristics of the UF group (case group) and non-fibroids (control group) are shown in **Table 1** and **Supplementary Figure S1**. There is no difference between the case and control groups in terms of BMI, menstrual history, previous history, and childbearing history, and a difference in average age was detected (*P* = 0.02) between the two groups.

**Table 1:** Characteristics of the study subjects.

Variables	Uterine fibroid group (*n* = 43)	Control group (*n* = 42)	*P*-value	
Age [year, median (range)]	43 (24–52)	35 (23–54)	0.02	
BMI [kg/m^2^, (mean ± SD)]	23.34 ± 3.22	22.48 ± 3.53	0.24	
Menstrual history, *n* (%)			0.07	
Increased	18 (41.86%)	10 (23.26%)		
No change	25 (58.14%)	33 (76.74%)		
Major previous history, *n* (%)				
PCSD	4 (9.30%)	10 (13.25%)	0.08	
Endometrial polyp	10 (23.26%)	17 (39.53%)	0.10	
Number of gravidities [median (range)]	3 (0–7)	2 (0–8)	0.28	
Number of parities [median (range)]	1 (0–4)	1 (0–3)	0.32	
Number of abortions [median (range)]	1 (0–6)	1 (0–5)	0.26	
Modes of delivery, *n* (%)			0.30	
Spontaneous delivery	26 (74.29%)	20 (62.50%)		
Menstruation				
Duration [day, median (range)]	6 (4–12)	7 (5–12)	0.06	
Frequency [day, median (range)]	28 (24–45)	28 (22–40)	0.29	

BMI, body mass index; SD, standard deviation; PCSD, previous cesarean scar defect.

### The Diversity of the Gut Microbiota

**Figure 1:**
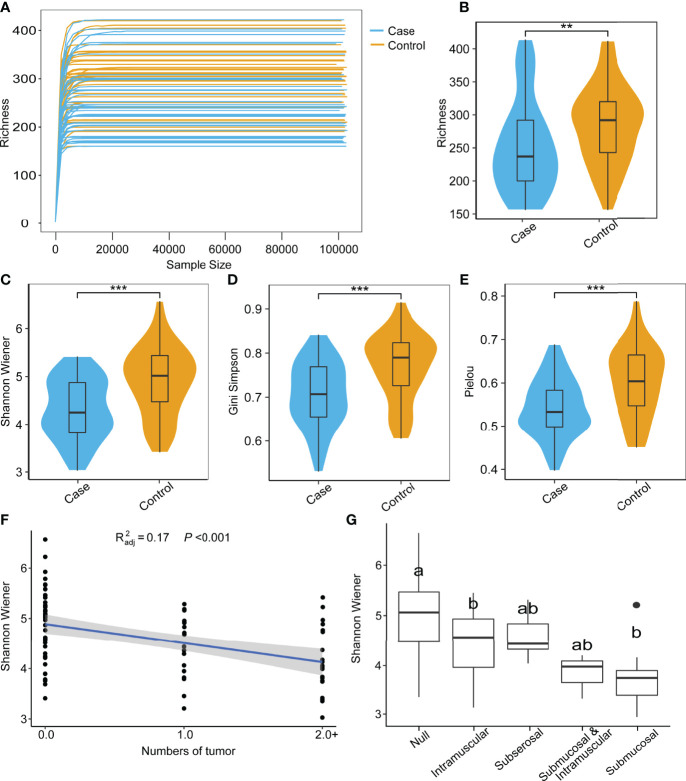
Species rarefaction curves and alpha diversity of microbial communities. **(A)** Species rarefaction curves of 85 samples. Alpha diversity index between case and control groups: richness **(B)**, Shannon–Wiener **(C)**, Gini-Simpson **(D)**, and Peilou **(E)** (Wilcoxon test, **P < 0.01, ***P < 0.001). **(F)** Regression analysis of tumor number and Shannon–Wiener (Wilcoxon test). **(G)** Comparison of Shannon–Wiener among different locations of tumor occurrence (ONE Tukey HSD).

**Figure 2:**
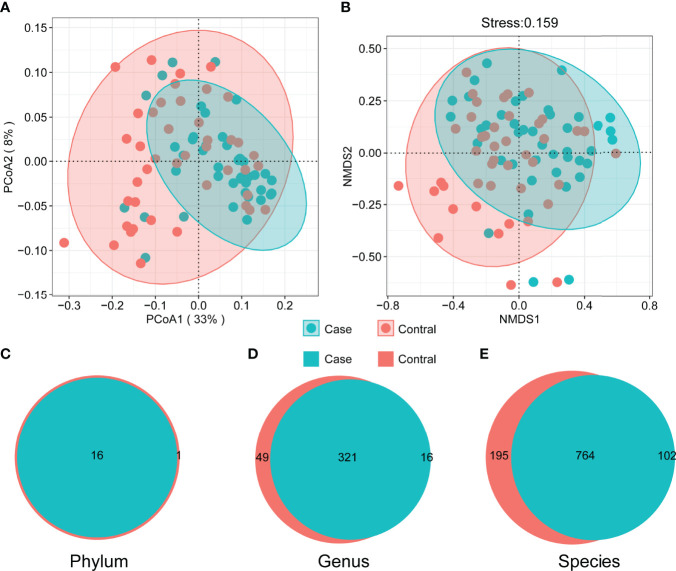
Beta diversity of microbial communities. Principal coordinate analysis **(A)** and non-metric multidimensional scaling **(B)** (ANOSIM *R* = 0.089, *P* = 0.001). The shaded area marks 95% confidence interval. The difference of composition in phylum **(C)**, genus **(D)**, and species **(E)** level between two groups.

All samples were sequenced to sufficient depth and dilution curves, which were calculated and recorded after 5 replicate random samples (**Figure 1A**). The control group had higher indices than the case group in terms of all α-diversity indices: richness (**Figure 1B**), Shannon–Wiener (**Figure 1C**), Gini–Simpson (**Figure 1D**), and Pielou (**Figure 1E**). β-Diversity was compared using both the algorithm of NMDS and principal coordinate analysis (PCoA), which demonstrate significant differences between the two groups. The PCoA results showed that the distribution of cases and controls was scattered between groups and clustered within groups with a smaller area in the case group and the ANOSIM test (*P* = 0.001). The difference between groups was greater than that within groups, implying a significant difference in diversity between the case and control groups (**Figure 2A**). The NMDS analysis results were similar to those of PCoA and stress = 0.159 (<0.2) (**Figure 2B**). The α-diversity index of gut microbes was further investigated in patients with different number and locations of tumorigenesis. Interestingly, our results revealed that the gut microbial diversity of patients decreased with the increasing number of tumors (*P* < 0.01). Some differences were also observed in gut microbial α-diversity indices depending on the location of tumorigenesis (**Figures 1F, G** and **Supplementary Figure S2**).

### The Composition and Biomarkers of the Gut Microbiota

**Figure 3:**
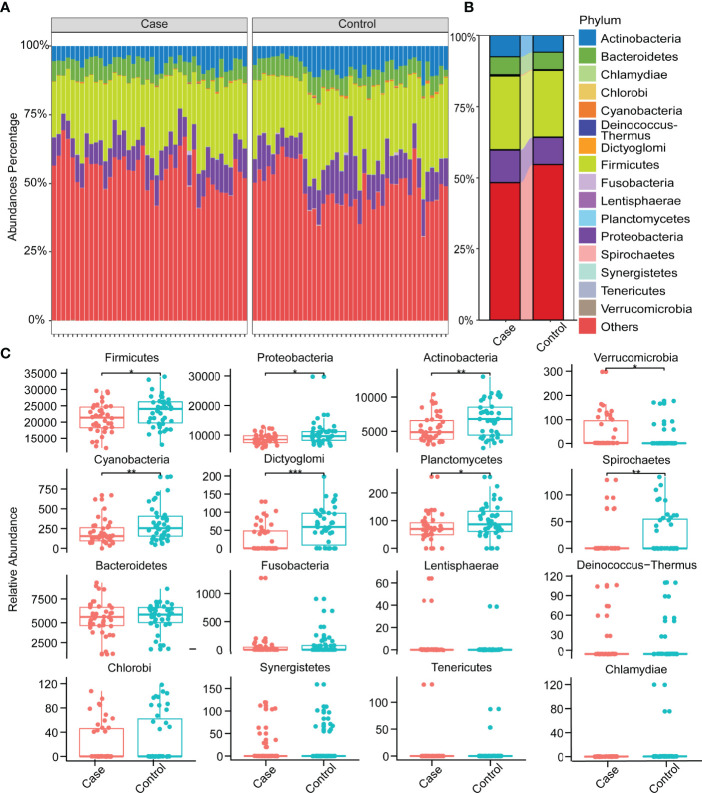
Relative abundances of species at the phylum level. **(A)** Relative abundances of species at the phylum level in all samples. **(B)** Relative abundances in phylum level in two groups. **(C)** Distribution of all species at the phylum level and differences between groups (Wilcoxon test, *P < 0.05, **P < 0.01, ***P < 0.001).

**Figure 4:**
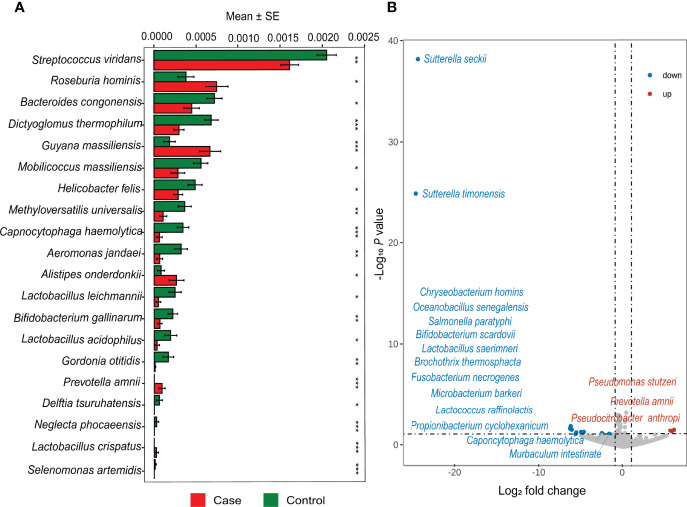
Differential abundance analysis at the species level. **(A)** Top 20 different relative abundance species between case and control based on Metastats. (*P < 0.05, **P < 0.01, ***P < 0.001) **(B)** The volcano map shows significant upregulation and downregulation in the case group compared with the control group.

There were commonalities and differences in species composition between the case and the control groups. At the phylum level, 16 phyla were in the case group, while 17 phyla were in the control group (**Figure 2C**). At the genus level, 337 genera were in the case group, 370 were in the control group, and 321 were common to both groups (**Figure 2D**). In total, 866 species in the case group and 959 in the control group were at the species level. Among them, 764 were common, while 195 were unique to the control group and 102 to the case group (**Figure 2E**). In order to demonstrate the differences in taxonomy composition between the case and the control groups, we compared the differentially expressed species between the two groups at the phylum and the species level, respectively. The results suggest that the composition of the fractions was similar, but the abundance percentage of the components varied at the phylum level (**Figures 3A, B**). Specifically, the relative abundance of Firmicutes, Proteobacteria, Actinobacteria, Cyanobacteria, Dictyoglomi, and Spirochaetes, respectively, was significantly lower in the case group than in the control group (*P* < 0.05) (**Figure 3C**). Besides this, among all components, only Verrucomicrobia showed the opposite trend (**Figure 3C**). Furthermore, we analyzed the differential abundance of species based on statistical differences (Metastats), and the multiplicity of differences characterized the biomarkers (DESeq2). For Metastats analysis, we presented the top 20 differentially expressed species in relative abundance (*P* < 0.05) (**Figure 4A**). In total, 17 biomarkers were found, 3 of which (marked in red) were upregulated, while 14 (marked in blue) were downregulated in the case group relative to the controls (**Figure 4B**).

### The Microbial Interactions and Networks Between Gut Microbiotas

**Figure 5:**
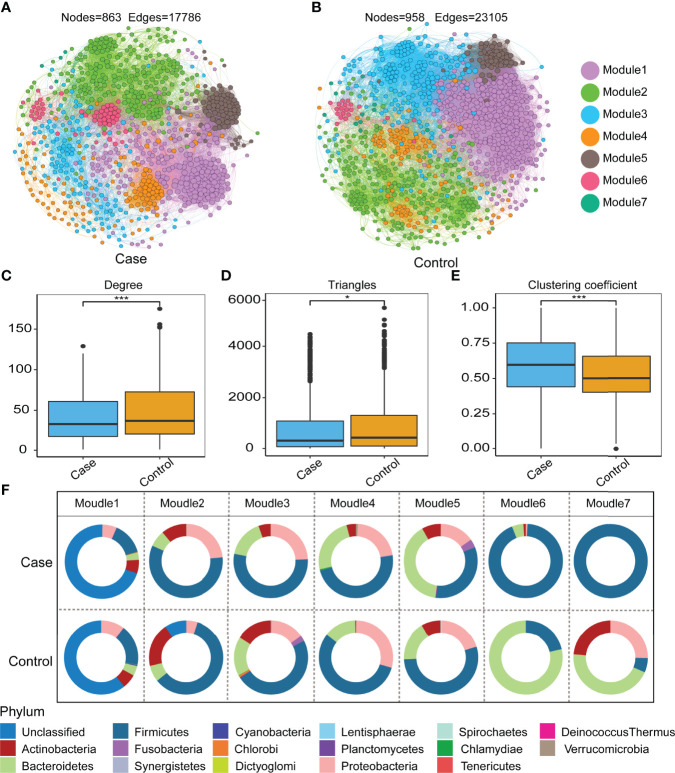
Co-occurrence network in the control and case groups. There are 7 modules in the case group **(A)**, while 7 groups are in the control group **(B)**. Topological features of the network: degree **(C)**, triangles **(D)**, and clustering coefficient **(E)** (Wilcoxon test, *P < 0.05, ***P < 0.001). Node connectivity (degree) shows how many connections (on average) each node has to the other nodes in the network. Triangles are the number of vertex triangles in a network diagram, reflecting connectivity. The global aggregation coefficient is a parameter that reflects the closeness of nodes in a network, also known as transferability. **(F)** The doughnut shows relative abundance in 7 modules at the phylum level between the two groups.

We performed a network co-occurrence analysis to unravel the relationships among microorganisms. The resulting case network consisted of 863 nodes linked by 17,786 edges, with a much higher number of strong positive correlations (17,311, 97.33%) than negative ones (475, 2.67%), and the average number of edges per node was 2.978. The control network consisted of 958 nodes linked by 23,105 edges, also with a much higher number of strong positive correlations (21,665, 93.77%) than negative ones (1,440, 6.23%), and the average number of edges per node was 2.798 (**Supplementary Table S1**). The results suggest that microbial networks were made up of closely connected nodes and formed a kind of “small world” topology (**Supplementary Table S1**). Compared with the topological properties of the random network with the same number of nodes and edges (**Supplementary Figure S3** and **Supplementary Table S2**), the network of the case group exhibited a scale-free characteristic (*P* < 0.001, **Supplementary Figure S4**), and the control group also exhibited a scale-free characteristic (*P* < 0.001, **Supplementary Figure S5**), indicating that the network structure was non-random. Both the gut microbial interaction networks of the case group and the control group were divided into seven modules (**Figures 5A, B****)**. The average degree of the case group is 41.219, which is lower than that of the control group (48.236, *P* < 0.001, **Figure 5C**), and the number of sides forming triangles is also lower (*P* < 0.05, **Figure 5D**). This suggests that the total connectivity and complexity between gut microbes was higher in the control group than in the case group. The average path length is 2.978 in the case group and 2.798 in the control group (**Supplementary Table S1**); the average clustering coefficient of the case group is 0.593, which is significantly higher than that of the control group (0.525, *P* < 0.001, **Figure 5E**). These results manifest that the average “clustering property” of the whole network between gut microorganisms in the case group was higher than that in the control group. We drew a doughnut to show the taxonomy composition of each module of the case and the control group networks at the phylum level (**Figure 5F**). The results show that a difference in the components and the abundance percentage exists in each module. Given the above-mentioned findings, it can be concluded that there are differences in the gut microorganism interaction network between the case group and the control group. Compared with the control group, the case group network has lower connectivity and complexity and higher clustering property.

## Discussion

It was found that gut microbiome in UFs was altered in composition, ecological network, and functionality compared with healthy women. We identified the differences of UF group in gut microbiota, also explored the potential correlation, and deciphered the interplay between the gut microbiome and UFs. The α-diversity in patients with UFs was significantly lower than that of healthy controls and negatively correlated with the number of tumorigeneses. The microbial composition of the UF patients deviated from the cluster of healthy controls. Stool samples from patients with UFs exhibited significant alterations in terms of multiple bacterial phyla, such as Firmicutes, Proteobacteria, Actinobacteria, and Verrucomicrobia. In differential abundance analysis, some bacteria species were shown to be downregulated (*e*.*g*., *Bifidobacteria scardovii*, *Ligilactobacillus saerimneri*, and *Lactococcus raffinolactis*) and upregulated (*e*.*g*., *Pseudomonas stutzeri* and *Prevotella amnii*). Furthermore, the microbial interactions and networks in UFs exhibited lower connectivity and complexity as well as higher clustering property compared to controls.

Imbalance in gut microbiota composition is associated with a series of non-communicable diseases, including gastrointestinal disorders (inflammatory bowel diseases, liver cancer, colorectal cancer), metabolic diseases (type 2 diabetes, obesity, malnutrition, atherosclerosis, metabolic liver disease), and neurodegenerative diseases (Alzheimer’s disease, Parkinson’s disease) (Addolorato et al., 2020; Kim et al., 2020), all of which are characterized by a decreasing microbial diversity. In our study, the α-diversity of the gut microbiota in the control group was significantly higher than that of the case group (*P* < 0.01). In addition, the PCoA analysis of microbiota composition indicated that there was a distinct clustering pattern between samples from UF individuals and healthy controls. These results were also in line with previous research on reproductive endocrine and metabolic disorders, which found that the alpha diversity in polycystic ovary syndrome was lower than that in healthy people (Qi et al., 2019; Jobira et al., 2020; Jiang et al., 2021). Interestingly, in our study, the alpha diversity of microbiota was negatively correlated with the number of tumorigeneses. However, further experiments are needed to verify and explore the possible mechanisms in benign UFs. Taken together, these observations may indicate that a low level of richness and evenness may lead to gut flora dysbiosis, which is associated with increased risk for UFs in women. However, some microbiome studies on endometriosis, a sex hormone-related disease, showed different alterations (Yuan et al., 2018; Ata et al., 2019).

By analyzing differential abundance, we observed that the upregulated species were *Prevotella amnii* and *Pseudomonas stutzeri*, while the downregulated species were *Lactobacillus saerimneri* and *Lactococcus raffinolactis* among UF patients. *Prevotella amnii* was reported to be enriched in patients with breast cancer, as it was involved in regulating or responding to host immunity and metabolic balance (Zhu et al., 2018). *Pseudomonas stutzeri* is widely distributed in natural environments, and this species could be considered an opportunistic pathogen that is more abundant in bone and urinary tract infections, especially in patients with acquired immune deficiency syndrome (Lalucat et al., 2006). Moreover, *Lactococcus raffinolactis* is associated with aldehyde dehydrogenase, an alcohol metabolism-related enzyme, and this species has the potential to be a promising dietary supplement probiotic (Konkit et al., 2016). Our finding was in line with previous research which suggested that *Lactobacillus saerimneri* had higher relative abundance in a healthy and younger population and was associated with potent tumor necrosis factor-inhibitory activity (Ma et al., 2021). In other words, our study showed a significant decrease in probiotics and an increase in pathogenic bacterial species among UF subjects, indicating their reduced ability to maintain homeostasis and the increased risk of disease.

The ecological network of gut microbiota is considered critical to host health because it indicates that beneficial symbionts and their associated functions are maintained over time (Lozupone et al., 2012; Relman, 2012). Dysbiosis of the intestinal microbiota is reflected not only at the level of changes in the abundance of flora members but also in the altered relationships of microbial interactions (Chen et al., 2020). Our network analysis demonstrated lower connectivity and complexity and higher clustering in the case group network compared to the control group. Microbial communities showing high cooperation were regarded as less stable compared with a competitive community (Coyte and Rakoff-Nahoum, 2019). The gut microbiome in Chinese women with UFs was altered in composition, ecological network, and functionality compared with healthy women. Associated factors for the prediction of UFs were also identified.

However, certain limitations of the present study should also be considered. Firstly, dietary characteristics, which are potential confounders, were not described (Gershuni et al., 2021). Secondly, no precise mechanism was involved in the present study, including host estrogen–gut microbiome axis, immune regulation, and metabolism. Thirdly, although a bare age difference should not be totally ignored, this confounder can be explained from data analysis and clinical practice. On one hand, RDA analysis showed that four factors (age, BMI, menses, and menstruation) accounted for less than 4.52% of the differences in community structure (**Supplementary Figure S1**). On the other hand, individuals with UFs always have a long-term follow-up history before surgical treatment on admission, which indicates that age was unlikely to have been a confounding factor in this cohort. Therefore, further studies can clarify whether the association is causal and whether dysbiosis leads to UFs or the disease leads to gut dysbiosis. Furthermore, the whole bowel microbial environment may not be provided or reflected by fecal microbiota, which is closely related to the systemic status, but sampling multiple sites in the human intestine is health-threatening and unethical. It is feasible to refine the inclusion and exclusion criteria.

In conclusion, our preliminary study provided distinct evidence on the imbalance of gut microbiota in UF patients. Our results can lay the foundation for subsequent studies on microbiota biomarkers to predict UF risks. Additionally, the alterations may be used to guide the development of probiotic supplements that alleviate gut dysbiosis in UFs.

## Data Availability Statement

The microarray data reported in this paper have been deposited in the Gene Expression Omnibus 291 (https://www.ncbi.nlm.nih.gov/geo/), under accession number GSE197904.

## Ethics Statement 

The studies involving human participants were reviewed and approved by The Third Xiangya Hospital of Central South University and performed under the relevant guidelines and regulations (IRB number 22003). The patients/participants provided their written informed consent to participate in this study.

## Author Contributions

DX and ZY conceived the study. XM and XP performed the experiments and analyzed the data. XP, XM, XZ, and QP wrote and edited the final manuscript. All authors contributed to the article and approved the submitted version.

## Funding

This work was supported by the National Science Foundation of China (number 32000054 and number 32170071), the Natural Science Foundation of Hunan Province (number 2021JJ40956), and the Key Research and Development Program of Hunan Province (grant number 2018SK2102).

## Conflict of Interest

The authors declare that the research was conducted in the absence of any commercial or financial relationships that could be construed as a potential conflict of interest.

## Publisher’s Note

All claims expressed in this article are solely those of the authors and do not necessarily represent those of their affiliated organizations, or those of the publisher, the editors and the reviewers. Any product that may be evaluated in this article, or claim that may be made by its manufacturer, is not guaranteed or endorsed by the publisher.
