# Supplementary material for: Uterine Fibroid Patients Reveal Alterations in the Gut Microbiome
Source: Front Cell Infect Microbiol. 2022 May 11;12:863594. doi: 10.3389/fcimb.2022.863594 (PMC9131877; doi:10.3389/fcimb.2022.863594)
Supplement: Supplementary file 1 [file DataSheet_1.docx]

Supplementary Material

## Supplementary Figures


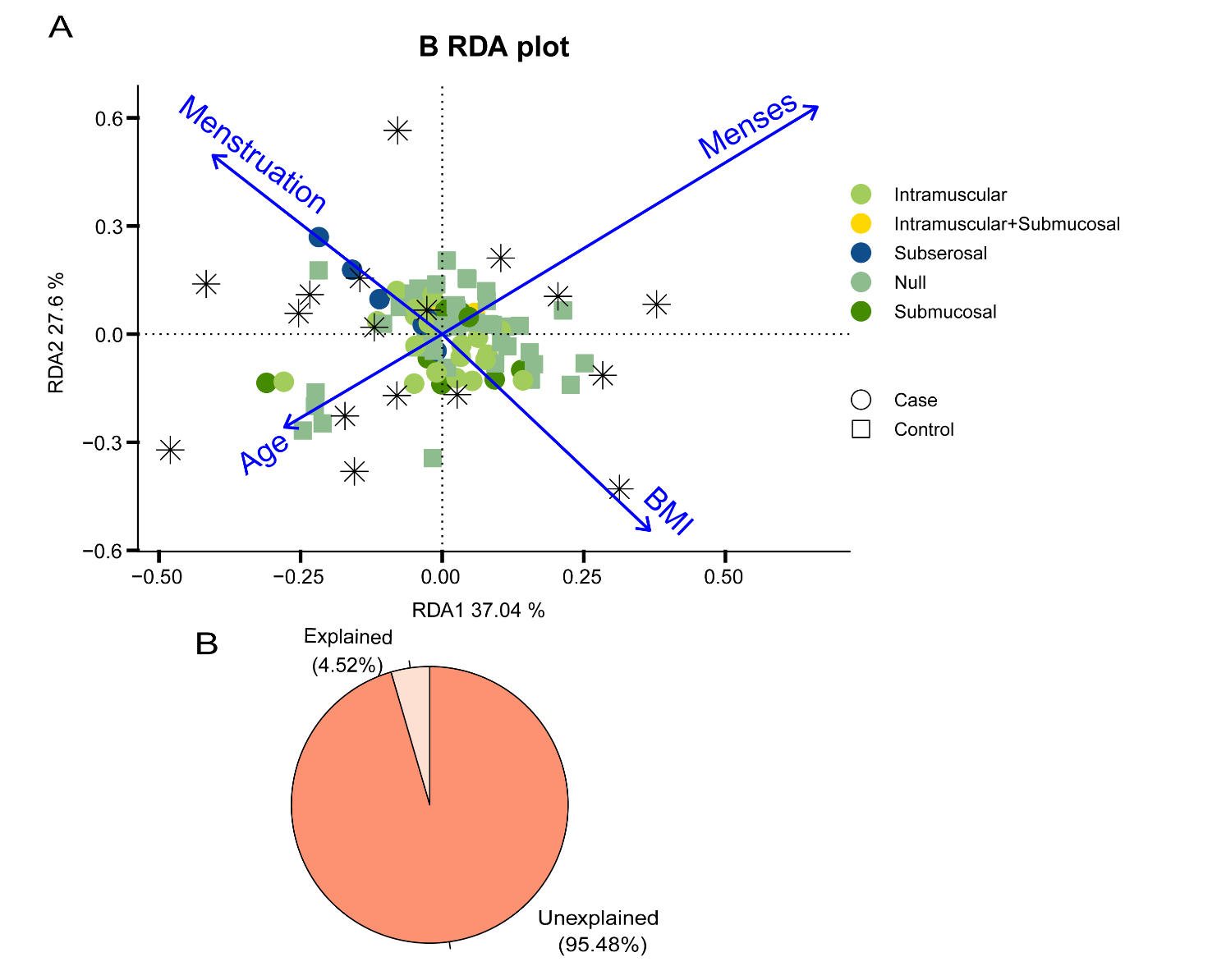


**Fig.S1** **(A)** RDA analysis of sample information, sample distribution, and community composition. The blue arrow shows sample information age, BMI, menses, and menstruation. Different shapes represent different groups. *Represents community composition at the phylum level. **(B)** The pie chart shows the explanation of sample information to the differences in community structure


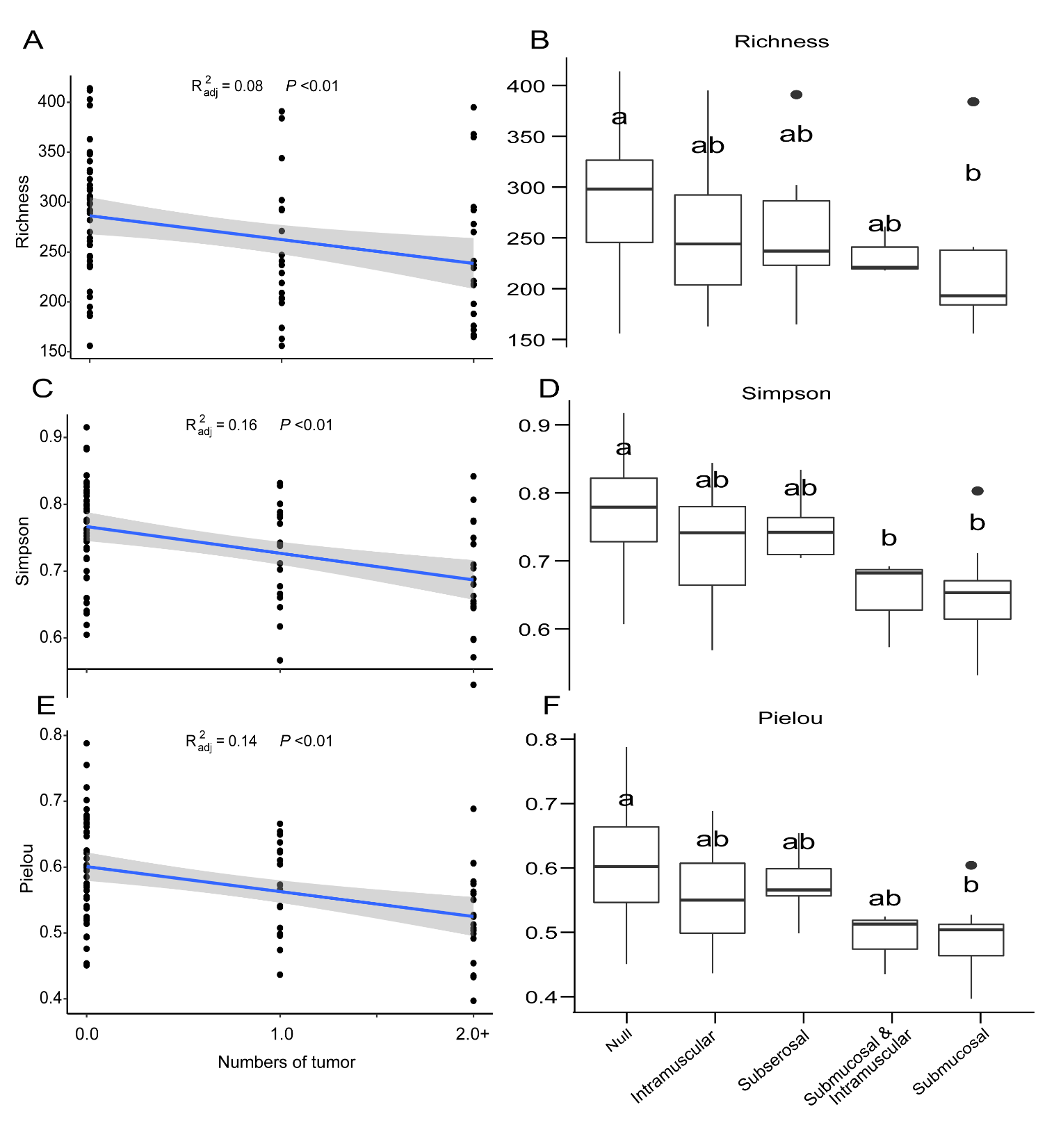


**Fig.S2** Regression analysis of tumor number and Richness **(A)**, Simpson(C), Pielou **(E)** (Wilcox. test). Comparison of Richness **(B)**, Simpson**(D)**, Pielou **(F)** among different locations of tumor occurrence (ONE Tukey HSD).


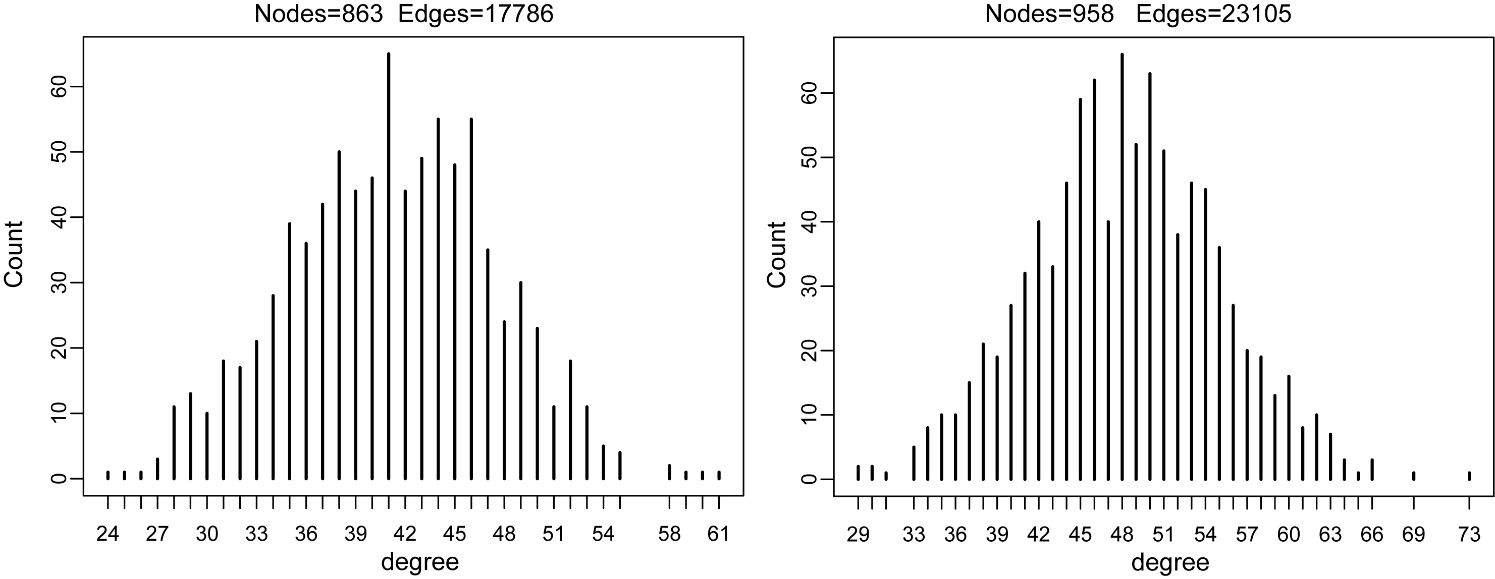


**Fig.S3** The distributions of degree for the associated random networks.


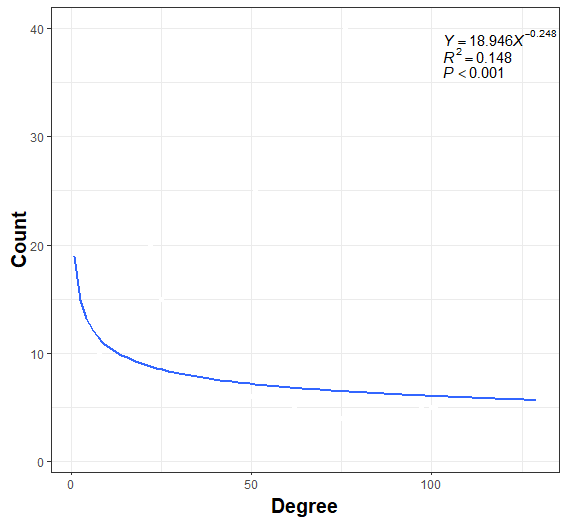


**Fig.S4** The distributions of degree for the case group’s real co-occurrence network. The *P-*value was obtained by randomly permuting the count values 999 times and obtaining the R^2^ (R^2^') from the data after randomly permuting the data. Comparing the frequency that R^2^' of the randomly permuted value is greater than the R^2^ of the observed value, that is, the *P-*value.


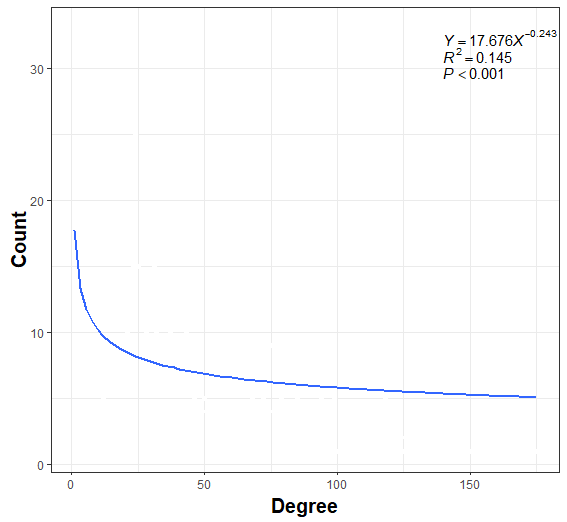


**Fig.S5** The distributions of degree for the control group’s real co-occurrence network. The *P-*value was obtained by randomly permuting the count values 999 times and obtaining the R^2^ (R^2^') from the data after randomly permuting the data. Comparing the frequency that R^2^' of the randomly permuted value is greater than the R^2^ of the observed value, that is, the *P-*value.

## Supplementary tables

**Table S1. The topological features of network**

|  | Nodes ^a^ | Edges^b^ | Modularity ^c^ | | Average clustering coefficient ^d^ | Network diameter ^e^ | Average path length ^f^ | Average degree ^g^ | | No. of  modules |
| --- | --- | --- | --- | --- | --- | --- | --- | --- | --- | --- |
| Case  Control | 863  958 | 17786  23105 | 0.706  0.573 | 0.593  0.525 | | 7  7 | 2.978  2.798 | | 41.219  48.236 | 7  7 |

Random networks were generated by rewiring all of the links with the same numbers of nodes and edges to the real networks

The number in the brackets indicates the standard deviation of topological properties of the 1000 Erdös-Rényi random networks

a Number of OTUs with the correlation *r* > 0.4 or *r* < –0.4 and statistical significance (*P* < 0.05)

b Number of strong and significant correlations between nodes

c Modularity >0.4 suggests that the network has a modular structure. It indicates that there are nodes in the network that are more densely connected between each other than with the rest of the network and that their density is noticeably higher than the graph’s average.

d How nodes are embedded in their neighborhood, and the degree to which nodes tend to cluster together

e The maximum distance between all possible pairs of nodes

f The average number of steps along the shortest paths for all possible pairs of network nodes

g Node connectivity showing how many connections (on average) each node has to the other nodes in the network

**Table S2 The associated random networks**

| Nodes ^a^ | Edges ^b^ | Modularity  (SD) ^c^ | Average clustering coefficient (SD)^d^ | Average length(SD) ^f^ | Average degree ^g^ |
| --- | --- | --- | --- | --- | --- |
| 863  958 | 17786  23105 | 0.123±0.002  0.112±0.002 | 0.047±0.000  0.050±0.000 | 2.085±0.000  2.033±0.001 | 41.219  48.235 |

Random networks were generated by rewiring all of the links with the same numbers of nodes and edges to the real networks

The number in the brackets indicates the standard deviation of topological properties of the 1000 Erdös-Rényi random networks

a Number of OTUs with the correlation *r* > 0.4 or *r* < –0.4 and statistical significance (*P* < 0.05)

b Number of strong and significant correlations between nodes

c Modularity >0.4 suggests that the network has a modular structure. It indicates that there are nodes in the network that are more densely connected between each other than with the rest of the network and that their density is noticeably higher than the graph’s average.

d How nodes are embedded in their neighborhood, and the degree to which nodes tend to cluster together

e The maximum distance between all possible pairs of nodes

f The average number of steps along the shortest paths for all possible pairs of network nodes

g Node connectivity showing how many connections (on average) each node has to the other nodes in the network
